# Microsurgical Anatomy of the Posterior Commissure and Habenular Commissure in the Human Cadaveric Brain

**DOI:** 10.3390/brainsci16050490

**Published:** 2026-04-30

**Authors:** Aysegul Esen Aydin, Mehmet Emin Akdeniz, Orhun Mete Cevik

**Affiliations:** 1 Department of Neurosurgery, Prof. Dr. Cemil Tascioglu City Hospital, University of Health Sciences, Istanbul 34384, Turkey; mehmetemin.akdeniz@sbu.edu.tr; 2 Department of Neurosurgery, Bakırköy Teaching and Research Hospital, Istanbul 34140, Turkey; orhunmete.cevik@saglik.gov.tr

**Keywords:** posterior commissure, habenular commissure, white matter fiber dissection, diffusion tractography

## Abstract

**Highlights:**

**What are the main findings?**

- Full-hemisphere lateral-to-medial fiber dissection preserves the three-dimensional architecture of the posterior and habenular commissures and demonstrates their distinct structural organization.
- The habenular commissure cannot be reliably reconstructed using deterministic tractography, despite high-density seeding and optimized parameters, yielding anatomically misleading anterior projections.

**What are the implications of the main findings?**

- A clear resolution gap exists between microsurgical anatomy and diffusion-based tractography for fine epithalamic commissural pathways.
- Cadaveric fiber dissection remains the reference standard for accurate delineation of midline commissural connectivity.
- Precise three-dimensional understanding of the PC–HC complex is essential for safe pineal and posterior third ventricular surgery.

**Abstract:**

Background/Objectives: The posterior commissure (PC) and habenular commissure (HC) are fine-caliber midline fiber bundles located within the epithalamic roof of the third ventricle. Their small size and deep anatomical position render them vulnerable during fiber dissection, and it is difficult to delineate reliably with conventional diffusion MRI. To define a reproducible microsurgical strategy for three-dimensional exposure of the PC and HC, and to evaluate their tractographic representation using high-resolution diffusion template data. Methods: Four formalin-fixed adult cadaveric brains were prepared using a modified Klingler technique and underwent systematic microsurgical fiber dissection focused on preservation of the epithalamic roof and midline commissures. Diffusion MRI data from the Human Connectome Project (HCP-1065) were reconstructed in MNI space using q-space diffeomorphic reconstruction in DSI Studio to attempt deterministic tractographic reconstruction of the PC and HC. Results: In all specimens, the PC was identified as a compact transverse bundle superior to the rostral cerebral aqueduct within the inferior pineal lamina. The HC appeared as a thinner band superior to the pineal recess, interconnecting the bilateral habenular nuclei and separated from the PC by the hypothalamic sulcus. A midline-prioritized dissection sequence facilitated preservation of commissural continuity. Deterministic tractography reproduced adjacent peduncular trajectories but failed to consistently reconstruct discrete HC or PC streamlines. Conclusions: Cadaveric fiber dissection remains the most reliable method for studying the fine commissural anatomy of the epithalamus. A midline-first, roof-preserving strategy enhances visualization of the PC and HC and may have implications for posterior third ventricular surgery and stereotactic targeting.

## 1. Introduction

The posterior commissure (PC) and the habenular commissure (HC) form a compact midline fiber complex within the dorsal diencephalon, at the interface of the pineal region, tectal plate, and posterior third ventricle. Although closely related in topographic location, these commissures differ substantially in their connectivity and functional significance. The PC has classically been associated with the pupillary light reflex and vertical gaze pathways, whereas the HC constitutes a delicate interhemispheric connection of the habenular nuclei and forms part of the dorsal diencephalic conduction system [1,2,3,4,5].

From a clinical perspective, this region is highly relevant because even subtle injury to the posterior commissural complex may result in disproportionate neurological consequences during pineal region and posterior third ventricular surgery. In particular, damage to the PC may contribute to vertical gaze dysfunction, while disruption of the epithalamic region may compromise anatomically and functionally significant habenular connections. In parallel, increasing interest in the lateral habenula as a potential neuromodulation target in treatment-resistant psychiatric and pain disorders has further heightened the need for precise anatomical delineation of the epithalamic commissural region [4,5,6,7].

Despite this importance, the microsurgical anatomy of the HC remains insufficiently illustrated in the literature compared with more widely recognized midline commissural structures. Most available descriptions are either embedded within broader anatomical accounts of the pineal or epithalamic region, or inferred indirectly from radiological and tractographic studies. However, fine-caliber commissural systems such as the HC and the small transverse fibers of the PC remain particularly challenging to evaluate on diffusion-based imaging because of their deep location, limited diameter, complex fiber angulation, and susceptibility to partial-volume effects [6,7]. Consequently, the relationship between anatomical ground truth and tractographic representation in this region remains incompletely resolved.

Cadaveric white matter dissection offers a unique opportunity to preserve and directly visualize the three-dimensional arrangement of these delicate midline pathways. Nevertheless, standard half-brain or medial-to-lateral dissections may interrupt commissural continuity and limit appreciation of their spatial organization. A full-hemisphere lateral-to-medial dissection strategy may therefore provide a more suitable anatomical framework for demonstrating the HC–PC complex while maintaining its relationship to surrounding ventricular, thalamic, and pineal structures.

Accordingly, the present study aimed to define a reproducible microsurgical strategy for exposing the PC and HC in the human cadaveric brain and to characterize their anatomical relationships through stepwise fiber dissection. In addition, we sought to compare these microsurgical findings with deterministic tractographic reconstruction using a high-resolution diffusion template in order to examine the extent to which current diffusion-based methods can reliably represent fine epithalamic commissural anatomy. By combining cadaveric dissection with tractographic analysis, this study seeks not only to document the microanatomy of the posterior commissural region but also to highlight the persistent resolution gap between direct anatomical observation and contemporary diffusion imaging in small midline fiber systems.

## 2. Materials and Methods

### 2.1. Microsurgical White Matter Dissection Technique

Four adult human cadaveric brains were included in this study. The specimens were obtained from an institutional anatomical laboratory and were processed in accordance with established protocols for postmortem neuroanatomical research. Due to anonymization procedures and institutional limitations, detailed demographic data—including exact age, sex distribution, brain weight, and postmortem interval (PMI)—were not available for all specimens.

All brains were macroscopically examined prior to inclusion and confirmed to be free of gross intracranial pathology or structural abnormalities. The use of structurally preserved specimens was considered sufficient for the qualitative microanatomical objectives of the present study.

All specimens were fixed in 10% formalin solution for a minimum of three weeks prior to dissection. Following fixation, each brain was immersed in running tap water and subsequently frozen at −16 °C for one week in accordance with the classical Klingler fiber dissection technique. Before dissection, the specimens were gradually thawed under warm running water for several hours to facilitate separation of myelinated fiber tracts and to minimize tissue fragility.

Microsurgical dissections were performed under a Carl Zeiss OPMI surgical microscope (Carl Zeiss Meditec AG, Oberkochen, Germany) at magnifications ranging from ×6 to ×40. Dissection proceeded in a systematic stepwise manner from lateral to medial, with the primary aim of preserving the anatomical continuity of midline commissural structures. This full-hemisphere dissection strategy was specifically adopted to avoid interruption of commissural fibers, which may occur in conventional medial-to-lateral or partial hemisphere approaches.

The pineal region and the posterior wall of the third ventricle were meticulously exposed to identify the PC and the HC. At each stage, high-resolution digital photographs were captured using a Canon EOS 77D camera (Canon Inc., Tokyo, Japan) equipped with a Canon EF 100 mm f/2.8 L IS USM macro lens (Canon Inc., Tokyo, Japan). The spatial relationships of the commissures to surrounding anatomical structures were systematically documented. The study was conducted in accordance with institutional ethical standards and complied with the principles of the Declaration of Helsinki for postmortem anatomical research.

### 2.2. Fiber Tracking

To correlate the white matter relationships observed in microsurgical dissection, tractographic analysis was performed using DSI Studio (Hou version; 30 January 2026, National Taiwan University, Taipei, Taiwan) [8] based on diffusion MRI data from the Human Connectome Project (HCP-1065) template [9]. The dataset includes high-quality diffusion data from 1065 healthy adult subjects (age range: 22–35 years), reconstructed in Montreal Neurological Institute (MNI) space using q-space diffeomorphic reconstruction to obtain the spin distribution function [10,11]. The final isotropic resolution of the reconstructed data was 1.25 mm.

A deterministic fiber-tracking algorithm (fourth-order Runge–Kutta [RK4]) was employed. The choice of a deterministic approach was deliberate and based on the need for strict anatomical control and direct comparability with cadaveric dissection findings. In contrast to probabilistic methods, which may increase sensitivity in regions of complex fiber architecture, deterministic tractography provides a more constrained and anatomically conservative reconstruction, reducing the likelihood of false-positive streamlines—particularly in small-caliber commissural systems.

Given the submillimetric dimensions, high curvature, and midline crossing characteristics of the epithalamic commissures, tracking parameters were optimized to maximize sensitivity while maintaining anatomical plausibility. An anisotropy threshold of 0.01 and an angular threshold of 90° were selected to allow for streamlined propagation in regions of low anisotropy and sharp fiber angulation. The step size was reduced to 0.50 mm to improve tracking resolution of small-diameter bundles. Minimum and maximum streamline lengths were set to 5 mm and 100 mm, respectively.

**Figure 1:**
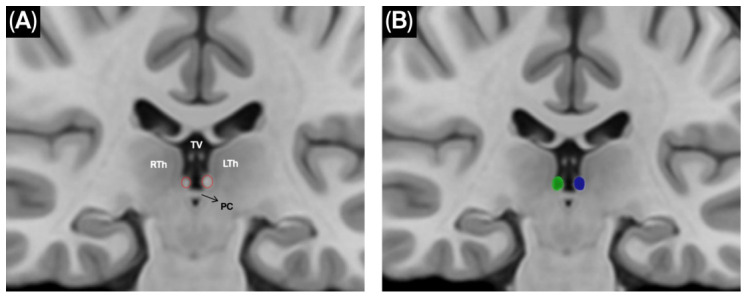
**Manual delineation of the bilateral habenulae on the HCP-1065 template.** (**A**) Coronal template view demonstrating the epithalamic region and key anatomical landmarks used to localize the habenulae. The right and left habenulae are highlighted by red dotted outlines. Abbreviations: RTh, right thalamus; LTh, left thalamus; PC, posterior commissure; TV, third ventricle. (**B**) Corresponding ROI placement for tractography on the same T1-weighted template background. The manually delineated ROIs are shown as the right habenula (green) and the left habenula (blue).

Because the habenular nuclei were not available as predefined labels in the default atlases, bilateral habenular regions of interest (ROIs) were manually delineated on the template using anatomical landmarks derived from the dissection planes (Figure 1). To ensure comprehensive exploration of potential fiber trajectories, seeding density was progressively increased from 1,000,000 to 10,000,000 seeds.

To minimize anatomically implausible reconstructions—particularly spurious anterior projections toward the anterior commissure—a coronal region of avoidance (ROA) was strategically placed between the habenular ROIs and the anterior commissural complex during targeted exclusion runs. Tractography results were qualitatively assessed through direct comparison with microsurgical dissection findings and by evaluating anatomical congruence of reconstructed streamlines with known epithalamic pathways.

Although probabilistic approaches—such as constrained spherical deconvolution (CSD)-based tractography—may offer advantages in resolving complex fiber configurations, the present study was specifically designed to test whether conventional deterministic tractography can reliably reproduce the fine commissural anatomy demonstrated by cadaveric dissection. This approach allows a more controlled evaluation of the limitations of diffusion-based connectivity in small midline fiber systems.

## 3. Results

### 3.1. General Organization and Orientation of Microsurgical Dissection

**Figure 2:**
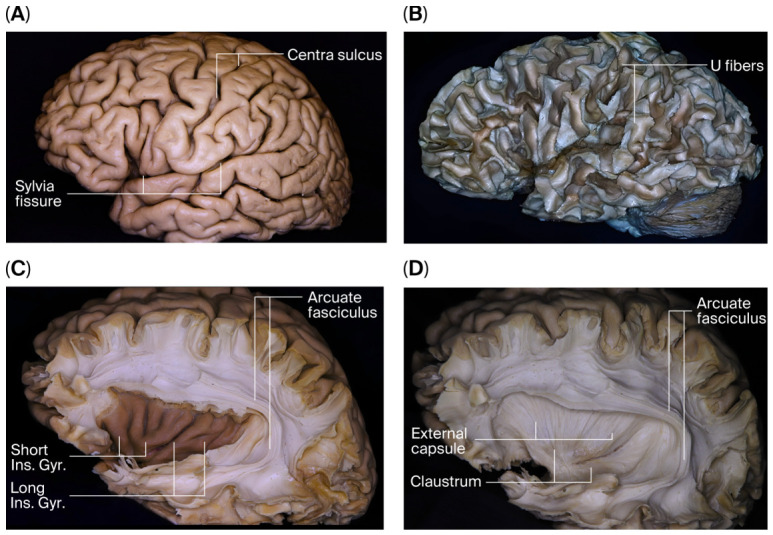
**Stepwise lateral-to-medial microsurgical fiber dissection of the cerebral hemisphere.** (**A**) Lateral surface of the cerebral hemisphere prior to initiation of dissection. (**B**) Removal of the cortical mantle of the frontal, parietal, temporal, and occipital lobes exposes the subcortical U-fibers. (**C**) Following removal of U-fibers, the superior longitudinal fasciculus is identified, demonstrating its relationship with the arcuate fasciculus and the insular cortex. (**D**) After resection of the arcuate fasciculus and insular cortex, the extreme capsule is exposed; its removal reveals the external capsule and the claustrum medially. Solid lines indicate anatomical boundaries, whereas dashed lines represent dissection planes.

Systematic microsurgical dissection was performed on four human cadaveric brains. The lateral surfaces of the cerebral hemispheres were initially identified macroscopically (Figure 2A). Dissection was planned from lateral to medial to preserve the anatomical integrity of the commissural complex, and the same sequence was followed in all specimens.

The cortical surfaces of the frontal, parietal, temporal, and occipital lobes were sequentially removed, exposing the underlying U-fibers (Figure 2B). The superior longitudinal fasciculus, located deep to the U-fibers, was dissected and removed. At this stage, the anatomical relationships of the arcuate fasciculus and the insular cortex were clearly demonstrated (Figure 2C). Following removal of the arcuate fasciculus and insular cortex, the extreme capsule was excised, revealing the external capsule and claustrum medially (Figure 2D).

**Figure 3:**
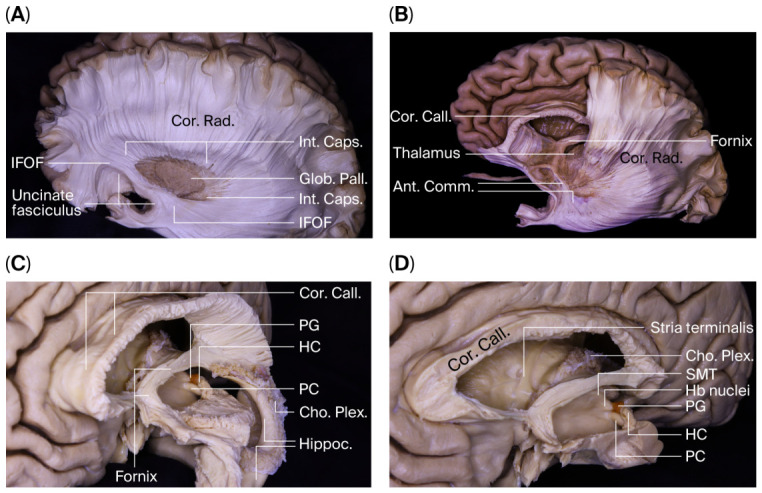
**Progressive exposure of deep hemispheric, ventricular, and epithalamic structures during full-hemisphere lateral-to-medial dissection.** (**A**) Following removal of the putamen, the internal capsule (Int. Caps.) and globus pallidus (Glob. Pall.) are exposed beneath the corona radiata (Cor. Rad.). Deeper dissection reveals the inferior fronto-occipital fasciculus (IFOF) and uncinate fasciculus, illustrating the laminar organization of hemispheric white matter. (**B**) Posterior to the corpus callosum (Cor. Call.), the fornix and its relationship with the ventricular roof are visualized. The anterior commissure (Ant. Comm.), thalamus, and surrounding projection fibers are identified within the context of the lateral ventricle. (**C**) Intraventricular exposure of the posterior third ventricle demonstrates, in a superior-to-inferior sequence, the suprapineal recess, pineal gland (PG), habenular commissure (HC), pineal recess, posterior commissure (PC), and cerebral aqueduct. (**D**) Detailed view of the epithalamic region showing the stria medullaris thalami (SMT), habenular nuclei (Hb nuclei), pineal gland (PG), habenular commissure (HC), posterior commissure (PC), and choroid plexus (Cho. Plex.), highlighting the spatial relationships between limbic pathways and the posterior wall of the third ventricle. Abbreviations: Cor. Call., corpus callosum; Cor. Rad., corona radiata; Int. Caps., internal capsule; Glob. Pall., globus pallidus; IFOF, inferior fronto-occipital fasciculus; Ant. Comm., anterior commissure; PG, pineal gland; HC, habenular commissure; PC, posterior commissure; SMT, stria medullaris thalami; Hb nuclei, habenular nuclei; Cho. Plex., choroid plexus.

After removal of the putamen, which lies medial to the external capsule, the globus pallidus and fibers of the internal capsule were exposed. With further deepening of the dissection, the inferior fronto-occipital fasciculus and uncinate fasciculus became distinctly identifiable (Figure 3A). This stepwise approach allowed a systematic demonstration of the laminar organization of hemispheric white matter.

### 3.2. Advancement to Deep Structures and Access to the Interhemispheric Region

Dissection progressed from the frontal lobe toward the parietal lobe and the interhemispheric fissure. Following the removal of the corona radiata fibers and the medially located tapetal fibers, the corpus callosum was visualized along the midline. It was identified in five anatomical subdivisions: rostrum, genu, body, isthmus, and splenium. Fibers arising from the genu and interconnecting the frontal lobes were recognized as the forceps minor, whereas fibers originating from the splenium and connecting posterior hemispheric regions corresponded to the forceps major.

Posterior to the corpus callosum, the fornical structures and their relationship to the roof of the third ventricle were identified. The septum pellucidum was examined in relation to the frontal horn and body of the lateral ventricle. The thalamus was evaluated within its anatomical context, including its adjacency to the body, atrium, and temporal horn of the lateral ventricle (Figure 3B).

At the level of the temporal horn, the head, body, and tail of the hippocampus were systematically exposed. The fimbria–choroidal fissure relationship was preserved, allowing identification of the inferior choroidal point. Opening of the choroidal fissure provided access to the ambient cistern. Following hippocampal dissection, connections between limbic structures and deep ventricular formations were assessed.

### 3.3. Stria Medullaris, Fornix, and the Epithalamic Complex

The stria terminalis was traced posteriorly along the roof of the temporal horn, demonstrating its continuity with the septal region and hypothalamus. The stria medullaris thalami, coursing along the superomedial surface of the thalamus, was identified, highlighting its anatomical relationship with the septal area and habenula.

The crura of the fornix followed a characteristic C-shaped trajectory around the pulvinar, converging at the atrial level to form the body of the fornix. At the level of the foramen of Monro, the fornix was divided into columns that descend toward the mammillary bodies.

At this stage, the posterior wall of the third ventricle was evaluated in its anatomical entirety. From an anterior intraventricular perspective, the following structures were identified in a superior-inferior sequence: the suprapineal recess, pineal gland, HC, pineal recess, PC, and the cerebral aqueduct (Figure 3C).

### 3.4. Microanatomical Characterization of the Posterior and Habenular Commissures

The PC was identified as a compact transverse fiber bundle located on the posterior wall of the third ventricle, adjacent to the dorsal aspect of the cerebral aqueduct. A subset of its fibers appeared to originate from the posterior thalamic region and the level of the superior colliculus, coursing in association with the medial longitudinal fasciculus. The superior colliculi were observed inferoposterior to the PC.

Immediately superior to the PC, the HC was identified as a thinner and more anterior–superiorly positioned fiber bundle interconnecting the bilateral habenular nuclei. The HC was located in the superomedial region of the thalamus in both hemispheres and showed a more delicate fiber organization than the PC. The hypothalamic sulcus was observed to form an anatomical boundary between the two commissures.

Complete hemispheric dissection performed from lateral to medial enabled preservation of commissural fiber continuity with the contralateral hemisphere. Compared with a medial-to-lateral half-hemisphere approach, this technique allowed uninterrupted visualization of the three-dimensional organization of both the HC and PC fibers. These findings indicate that the direction of dissection is a critical determinant in maintaining anatomical integrity during microsurgical exploration of commissural structures (Figure 3D).

### 3.5. Tractography Findings

**Figure 4:**
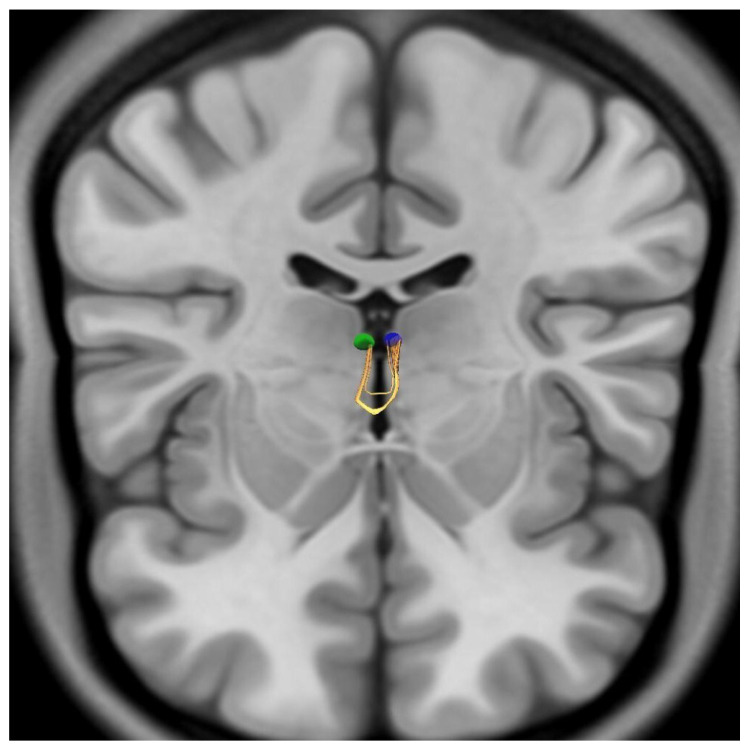
**Aberrant anteriorly projecting streamlines obtained from deterministic tractography of the habenular region.** Deterministic tractography performed using manually delineated bilateral habenular regions of interest (ROIs) failed to demonstrate a dorsal epithalamic crossing consistent with the habenular commissure (HC). Instead, reconstructed streamlines project anteriorly in a U-shaped configuration, converging toward and mimicking the anterior commissure, representing a dissection-incongruent trajectory. To reduce anatomically implausible projections, a region of avoidance (ROA) was placed at the level of the anterior commissure; however, no valid commissural streamlines connecting the bilateral habenular nuclei could be identified even at high seeding densities. Visualization is presented from an anterosuperior perspective with coronal and axial planes displayed. The right habenular ROI is shown in green, the left in blue, and reconstructed streamlines in yellow.

The tractographic analysis aimed to reconstruct the connectivity of the habenular region based on the anatomical coordinates derived from our dissections. Despite the application of high-density seeding and optimized parameters for small structures, the deterministic tractography failed to visualize the HC or the stria medullaris thalami as identified in our microsurgical specimens. Initial reconstructions, utilizing multiple seeding strategies between the manually delineated bilateral habenular nuclei, consistently yielded streamlines that followed an aberrant anterior projection. Instead of forming a dorsal epithalamic crossing, these fiber bundles projected anteriorly, merging with and anatomically mimicking the anterior commissure (Figure 4). To refine the results and eliminate these anterior artifacts, a Region of Avoidance (ROA) was implemented at the level of the anterior commissure. However, even with a sampling density of 10,000,000 seeds, no viable streamlines connecting the bilateral habenular nuclei across the posterior midline could be generated. This indicates a complete absence of a trackable signal for the inter-habenular connectivity within the 1.25 mm isotropic dataset under the specified constraints.

Deterministic tractography using the manually delineated bilateral habenular ROIs yielded a commissural-appearing trajectory that did not form a dorsal epithalamic crossing consistent with the HC or a course compatible with the stria medullaris thalami. Instead, the streamlines projected anteriorly in a U-shaped configuration and anatomically mimicked the anterior commissure, representing a dissection-incongruent reconstruction. The tractography is shown from an anterosuperior viewpoint with coronal and axial planes displayed (sagittal plane hidden). ROIs: right habenula (green), left habenula (blue). Streamlines are shown in yellow.

## 4. Discussion

The pineal recess, located in the posterior segment of the third ventricle, is bounded anteriorly by the HC and posteriorly by the PC. Although these two commissures are anatomically close, they are functionally distinct structures. The present study addresses a relevant gap in the literature by clearly delineating the PC–HC distinction at the dissection level and by demonstrating divergent fiber orientations for each commissure. Given the limited availability of human cadaveric images specifically illustrating the HC, the anatomical mapping presented here carries both clinical and educational significance [4].

### 4.1. Anatomical and Functional Framework of the Posterior Commissure

The PC is one of the earliest commissural fiber systems to develop during brain maturation and serves as a key landmark at the diencephalon–mesencephalon junction. It primarily consists of fibers interconnecting the pretectal nuclei, midbrain structures, and higher cortical regions involved in the pupillary light reflex [12]. The nuclei of Darkschewitsch and Cajal are closely associated with the PC. The nucleus of Darkschewitsch lies within the central gray matter at the upper end of the cerebral aqueduct, anterior to the oculomotor nucleus, whereas the interstitial nucleus of Cajal is situated near the superior aspect of the oculomotor fascicle. Both nuclei are critical for oculomotor coordination and vestibulo-ocular integration.

The PC is a principal commissural pathway responsible for the bilateral transmission of the pupillary light reflex. Consequently, PC lesions may result in vertical gaze palsy, light–near dissociation, and clinical syndromes such as Parinaud syndrome or dorsal midbrain syndrome [1,13,14].

Animal studies have suggested that certain PC fibers originate from the posterior thalamus and superior colliculus and continue directly into the medial longitudinal fasciculus. Fibers arising from thalamic, pretectal, and tectal regions—as well as from the superior colliculus and habenular nuclei—have been proposed to contribute to the PC [1]. However, our findings demonstrate clear differences in the course and organization of HC and PC fibers. Notably, the hypothalamic sulcus was identified as an anatomical boundary separating the two commissural systems.

The PC has been described as a dense and complex fiber network. It has also been reported to contribute to intertemporal connectivity through the sagittal stratum, which contains a combination of projection, commissural, and association fibers [15]. The sagittal stratum serves as a conduit linking the PC to regions involved in visual processing and higher cognitive functions. Among its components are the medial longitudinal fasciculus and the inferior fronto-occipital fasciculus [15,16].

Fiber dissection studies have further demonstrated that the PC establishes substantial connections with major white matter tracts [17,18]. These findings indicate that the PC plays an integrative role in maintaining functional coordination between the cerebral hemispheres while also interfacing with essential sensory and cognitive pathways. Continued advances in dissection and imaging techniques are refining our understanding of this complex commissural system.

### 4.2. Anatomical Characteristics and Clinical Relevance of the Habenular Commissure

The HC is a thin transverse white matter bundle within the epithalamus that interconnects the bilateral medial habenular nuclei. The habenular complex, a component of the dorsal diencephalic conduction system, consists of medial and lateral subdivisions and functions as a strategic relay between limbic forebrain structures and mesencephalic monoaminergic centers [4,19]. The structural integrity of this interhemispheric commissure provides the anatomical basis for coordinated bilateral habenular activity.

The principal afferent input to the habenular nuclei is conveyed by the stria medullaris thalami (SM), which carries projections from limbic and basal forebrain regions. Fibers originating from the septal nuclei, nucleus accumbens, and entopeduncular nucleus reach the habenular complex via the SM [20,21]. Diffusion tensor imaging studies have confirmed that the SM constitutes the major afferent white matter pathway to the lateral habenula [16]. Our anatomical findings are consistent with prior studies [22,23], and the relationship between the habenular nuclei and the SM was clearly demonstrated in our dissections.

The medial and lateral habenular subdivisions exhibit distinct connectivity patterns. The medial habenula primarily projects to the interpeduncular nucleus via the fasciculus retroflexus (habenulo-interpeduncular tract) and interacts indirectly with raphe nuclei [3,24]. In contrast, the lateral habenula establishes direct or indirect connections—via the rostromedial tegmental nucleus—with mesencephalic monoaminergic centers, including the ventral tegmental area, substantia nigra pars compacta, dorsal raphe nucleus, and median raphe nucleus [25].

This circuitry positions the lateral habenula as a critical node in dopaminergic and serotonergic modulation. Experimental studies have demonstrated that activation of the lateral habenula suppresses dopaminergic neuronal firing during reward omission and aversive expectation, contributing to the encoding of negative motivational value [25]. Structural alterations within the habenular complex have also been associated with affective disorders, particularly depression [19]. These observations suggest that disruption of habenular circuitry may contribute to maladaptive emotional regulation.

Efferent projections of the habenular complex are largely mediated by the fasciculus retroflexus, which transmits limbic inputs to mesencephalic output systems [20]. Through this bidirectional connectivity, the habenular complex assumes an integrative role in reward processing, stress responses, and affective regulation [4]. Disturbances in habenular connectivity have therefore been implicated in depression, anxiety, and treatment-resistant mood disorders.

### 4.3. Methodological Considerations: Dissection Versus Tractography

The discrepancy between microsurgical findings and tractographic reconstructions observed in the present study underscores a fundamental limitation in current diffusion-based imaging of fine diencephalic structures. While cadaveric dissection consistently demonstrated the habenular commissure (HC) as a distinct and continuous anatomical entity, deterministic tractography failed to reproduce this connectivity despite the use of high-density seeding and optimized tracking parameters.

This limitation is likely multifactorial. First, the small caliber of epithalamic commissural fibers places them at or below the spatial resolution threshold of conventional diffusion MRI datasets, including the 1.25 mm isotropic Human Connectome Project template used in this study. Partial-volume effects, particularly in regions containing cerebrospinal fluid and adjacent gray matter structures, may significantly attenuate directional anisotropy and impair streamline propagation [26,27,28]. Second, the sharp curvature and complex three-dimensional orientation of commissural fibers further challenge reconstruction algorithms and increase susceptibility to tracking bias [29,30]. The selection of a deterministic tractography approach in this study was intentional and methodologically driven. Deterministic algorithms provide a more constrained and anatomically conservative reconstruction framework, allowing direct comparison with microsurgical findings and minimizing false-positive streamlines that may arise in probabilistic approaches [29]. While probabilistic methods—particularly those based on constrained spherical deconvolution (CSD)—have demonstrated increased sensitivity in regions of complex fiber architecture, they may also introduce anatomically implausible pathways, especially in small and low-anisotropy fiber systems [29].

By applying a deterministic framework, the present study aimed not to maximize tract detection, but rather to critically evaluate whether conventional tractography can faithfully reproduce the fine commissural anatomy observed in cadaveric dissection. The inability to reconstruct the HC under these controlled conditions highlights a persistent resolution gap between microsurgical anatomy and diffusion-based connectivity models.

Future investigations employing ultra–high-field imaging (e.g., 7 Tesla MRI) and submillimetric acquisition protocols, in combination with advanced probabilistic reconstruction techniques, may improve visualization of these delicate structures [6]. Nevertheless, until such methods achieve consistent anatomical validation, cadaveric dissection remains the reference standard for the study of fine epithalamic commissural pathways [31].

### 4.4. Microsurgical Relevance and Conceptual Implications of the Habenular and Posterior Commissures

Beyond its descriptive anatomical scope, the present study introduces a methodological and conceptual framework for understanding the posterior commissure (PC) and habenular commissure (HC) as a continuous three-dimensional midline system. By employing a full-hemisphere lateral-to-medial dissection strategy, this study preserves commissural fiber continuity and enables direct comparison with tractographic reconstruction. This integrative approach demonstrates a consistent divergence between microsurgical anatomy and diffusion-based connectivity models in the epithalamic region, highlighting the limitations of current imaging techniques in resolving fine commissural pathways [26,27,28,29].

The pineal recess, located in the posterior segment of the third ventricle, is bounded anteriorly by the HC and posteriorly by the PC. Although these two commissures are anatomically close, they are functionally distinct structures. The HC primarily mediates interhemispheric connectivity between the medial habenular nuclei, whereas the PC is closely associated with pretectal and vertical gaze pathways, rendering it particularly sensitive in oculomotor control [4].

Although vertical gaze disturbances following PC injury are well recognized, specific neurological deficits attributable to isolated HC injury after pineal region surgery have not been clearly defined. During microsurgical dissection, advancement parallel to fiber orientation, minimal bipolar coagulation, and atraumatic manipulation of the pretectal region are recommended to preserve both anatomical and functional integrity.

In occipital transtentorial and supracerebellar infratentorial approaches, the direction of tentorial opening and depth of dissection may bring the surgical corridor into close proximity to the PC. Given its transverse fiber organization, the PC may be particularly susceptible to traction injury. In contrast, the HC, being thinner and located at the epithalamic roof, may be more vulnerable during narrow endoscopic approaches. In endoscopic supracerebellar infratentorial transpineal approaches to posterior-medial thalamic lesions, careful three-dimensional evaluation of commissural anatomy has been emphasized [32]. In this context, neuronavigation and intraoperative ultrasonography may contribute to preserving commissural boundaries.

The anterior commissure–posterior commissure (AC–PC) line remains a fundamental stereotactic reference. Although standardized in postmortem studies [33], high-resolution imaging has demonstrated interindividual variability in the angular relationship between AC and PC centers [7]. These findings suggest that commissures should not be regarded as fixed points, but rather as three-dimensional anatomical structures exhibiting individual variability.

Alternative interhemispheric connections through the PC have been described in cases of corpus callosum agenesis [34]. However, larger series have shown that such compensatory reorganization is inconsistent and varies among individuals [35]. From a surgical perspective, this variability implies that the functional consequences of commissural injury may not follow a predictable or uniform pattern.

Accordingly, preservation of the commissural complex during microsurgery may be structured around three core principles: controlled and symmetrical midline advancement; continuous assessment of the relationship between commissural fibers and venous structures; and dissection guided by fiber orientation, minimal traction, and low thermal impact.

In this context, the findings of the present study support a paradigm in which microsurgical anatomy and diffusion-based imaging should be interpreted as complementary rather than interchangeable representations of epithalamic connectivity. Integration of these approaches may be essential for achieving both anatomical accuracy and functional preservation in surgery of the pineal and posterior third ventricular region.

### 4.5. Limitations

This study has several limitations that should be acknowledged. First, the number of cadaveric specimens was limited to four, which may not fully capture the spectrum of interindividual anatomical variability. Although the overall configuration of the posterior and habenular commissures was consistent across all dissections, larger anatomical series would be required to assess potential morphometric differences and population-level variability.

Second, detailed demographic and postmortem data—including age, sex, brain weight, and postmortem interval (PMI)—were not available for all specimens due to anonymization procedures and institutional constraints. While such factors may influence tissue characteristics and preservation quality, all specimens were macroscopically confirmed to be free of gross intracranial pathology, and the study was designed as a qualitative microanatomical investigation rather than a morphometric analysis.

Third, the present study did not include quantitative morphometric measurements of commissural structures. Although such data would provide additional insight into spatial relationships and anatomical variability, the primary aim was to preserve and demonstrate three-dimensional fiber continuity through a full-hemisphere microsurgical dissection strategy.

Fourth, tractographic analysis was performed using a deterministic algorithm based on a population-averaged diffusion template with an isotropic resolution of 1.25 mm. While this approach enabled controlled comparison with microsurgical findings, it may underestimate the detectability of fine fiber systems. Advanced probabilistic reconstruction techniques and higher-resolution imaging modalities may improve sensitivity in future investigations.

Finally, although efforts were made to optimize figure clarity and anatomical correspondence in the revised version, the limited number of dissection images and perspectives may restrict full visualization of the regional complexity. Future studies incorporating multimodal visualization approaches may further enhance anatomical interpretation.

Despite these limitations, the combined use of full-hemisphere microsurgical dissection and tractographic analysis provides a consistent and anatomically grounded framework for understanding the posterior and habenular commissural complex.

## 5. Conclusions

This study delineates the three-dimensional microanatomical organization of the PC and HC within the midline using a lateral-to-medial full-hemisphere dissection strategy that preserves anatomical continuity. Our findings demonstrate that the direction of dissection is a critical determinant in maintaining commissural fiber integrity. In particular, half-hemisphere or medial-to-lateral approaches may limit comprehensive visualization of the HC and compromise its structural continuity.

Deterministic tractography failed to reliably reconstruct the HC despite high-density seeding and optimized tracking parameters. This suggests that fine caliber epithalamic commissural structures may not be adequately represented with conventional 1.25 mm isotropic diffusion data. These results highlight a persistent gap in resolution between microsurgical anatomy and diffusion-based connectivity analyses in small midline structures.

From a surgical perspective, the HC and PC should be regarded not only as topographic landmarks in pineal recess and posterior third ventricular surgery but also as functionally sensitive commissural pathways. Advancement parallel to fiber orientation, minimal traction, and individualized assessment of anatomical variation are essential principles for safe midline dissection.

Cadaveric dissection, therefore, remains the reference standard for morphological validation of the epithalamic commissural complex. Future submillimetric diffusion imaging and ultra–high-field magnetic resonance techniques may facilitate in vivo confirmation of the microanatomical relationships demonstrated through microsurgical dissection.

## Data Availability

No new datasets were generated or analyzed in this study. All data supporting the findings are included within the article. The diffusion MRI template used for tractography is publicly available from the Human Connectome Project (HCP) database.

## Abbreviations

The following abbreviations are used in this manuscript:

PC	Posterior commissure
HC	Habenular commissure
